# Supplementary material for: Are carbon emissions trading and green financial instruments synergistic? -Comprehensive quantitative research based on content analysis
Source: PLoS One. 2024 Mar 7;19(3):e0298601. doi: 10.1371/journal.pone.0298601 (PMC10919601; doi:10.1371/journal.pone.0298601)
Supplement: S1 Data — (ZIP) [file pone.0298601.s001.zip › Data/地级市Z_F工作报告.zip!/║╙▒▒/╩»╝╥╫»╩╨/└┤╫╘╣1⁄2╓┌║┼í╛┬φ┐╦╩2╛▌═°í┐_2017.docx]

各位代表：

现在，我代表市人民政府，向大会报告工作，请予审议，并请市政协委员和列席会议的同志提出意见。

一、过去四年工作回顾

市十三届人大一次会议以来，在党中央、国务院、省委、省政府和市委的正确领导下，市政府紧紧依靠全市人民，认真践行五大发展理念，积极融入京津冀协同发展，统筹推进稳增长、调结构、促改革、治污染、惠民生，经济社会发展取得了新成就，较好地完成了“十二五”规划确定的主要目标任务，实现了“十三五”良好开局，为全面建成小康社会奠定了坚实基础。

综合经济实力显著增强。全市生产总值由2012年的4500.2亿元增加到2016年的5857.8亿元，年均增长7.9%，占全省比重由16.9%提高到18.4%。全部财政收入、一般公共预算收入分别由573.4亿元、272.3亿元，增加到845.3亿元、410.8亿元，年均分别增长10.2%、10.8%。固定资产投资由3673.3亿元增加到5916亿元，投资总量连续四年位居全省第一。规模以上工业增加值、实现利润分别由1800.2亿元、562.7亿元增加到2190.3亿元、808.2亿元，利润总量保持全省首位。社会消费品零售总额由1915.8亿元增加到2975.2亿元，年均增长11.6%。城乡居民人均可支配收入分别达到30459元、12345元，比2012年增加8514元和3868元。

转型升级步伐持续加快。经济结构日趋合理，三次产业比重由10∶49.8∶40.2调整到8.2∶45∶46.8。供给侧结构性改革取得初步成效。累计压减炼钢产能260万吨、炼铁产能158万吨、水泥产能2983万吨，提前超额完成省下达的目标任务，市域内煤矿行业全部退出。规上工业综合能源消费量比2012年降低14.5%，单位增加值能耗比2012年下降39.3%。高新技术产业加快发展。高端装备制造等高新技术产业增加值完成382.6亿元，占规上工业增加值的比重达到17.5%，比2012年提高3.8个百分点；全市高新技术企业达到556家，比2012年翻了一番多。服务业发展全面提速。全市服务业增加值完成2738.9亿元，增长10.3%，总量跃居全省第一，对经济增长的贡献率达到69.7%，成为拉动经济增长的主要力量。现代农业加快发展。2016年粮食总产达到495万吨，连续三年被评为“全国粮食生产大市”。农业机械化率达到90.6%，农业产业化经营率达到66.8%，全市现代农业园区达到261个，成功获批国家现代农业示范区。创新驱动能力不断提高。市级以上创新型（试点）企业达到151家，科技型中小企业达到6193家，分别比2012年增加83家、5473家。省级以上科技企业孵化器达到15家，众创空间达到59家。石家庄高新区在全国综合排名从2012年的第38位上升至2016年的第15位，跻身全国“第一方阵”。

城乡面貌发生明显变化。行政区划调整顺利完成，城市发展空间进一步拓展。交通路网更加完善。京石武高铁投入使用。京港澳高速改扩建、京昆高速石太北线建成通车，南绕城高速、西阜高速、平赞高速全线开工，京赞线、正南线等主干公路顺利竣工。建成高速公路、国省干线78.3公里、农村公路5150公里，人民群众出行更加方便快捷。城市功能大幅提升。轨道交通1号线、3号线首开段空载试运行。胜利大街、新城大道等新改建工程竣工投用，南二环东西延、和平路高架桥西延等重大工程进展顺利。新建南水北调工程配套水厂22座，主城区实现引江水切换，日供水达到30万吨。成功入选国家地下综合管廊试点城市，启动实施总长45.7公里的地下综合管廊项目。建成投用市体育公园、滹沱河滨水公园。数字城管覆盖“八区一县”，精细化管理水平大幅提升。正定新区建设和古城保护加快推进。园博园正式开园，奥体中心主体完工，国际展览中心等5个大型功能性场馆开工建设，“三纵三横”路网框架初步形成，累计完成投资463亿元。正定古城保护十项重点工程顺利实施，“北方雄镇”历史风貌初步呈现。县城建设力度加大。努力开展县城建设攻坚行动，新增平山、元氏等5个省级园林县城，高邑创建成国家级园林县城。美丽乡村建设成效显著，累计完成1619个省级重点村改造任务。2016年全市常住人口城镇化率达到59.96%，比2012年提高6.46个百分点。

全面深化改革扎实推进。重点领域和关键环节改革取得突破性进展。“放管服”改革持续深化。累计取消下放和调整行政审批事项578项，非行政许可审批和行政监管类别全部取消。市、县两级行政审批局组建工作全部完成。“三证合一、一照一码”制度实现全覆盖，万人拥有市场主体数量达到688户，超过全国平均水平。财税金融改革取得新进展。有效落实积极的财政政策，“营改增”全面推开，水资源费改税工作全省领先，为企业减税120亿元以上。2016年新增贷款1051.2亿元，贷存比达到64.5%；在各类资本市场挂牌上市企业达到162家，比2012年增加129家。农村综合改革稳步推进。农村土地承包经营权确权登记基本完成，土地流转面积226.5万亩，走在全省前列。同时，国企国资改革、供销社综合改革等都取得积极进展。对外开放不断扩大。综合保税区正式开关运行。成功举办石洽会、冀商大会等招商活动。2016年实际利用外资12.2亿美元，增长7.1%；外贸进出口总值完成765.7亿元，占全省的比重达到24.9%。积极融入京津冀协同发展，引进京津合作项目253个，总投资4019.7亿元。

生态环境治理取得实效。持续推进大气污染防治，累计压减燃煤1084万吨，淘汰燃煤锅炉3200台，完成散煤替代60万吨，淘汰黄标车20.14万辆，整治露天生产矿山114座，搬迁主城区污染企业12家，提前一年完成“十二五”总量减排任务。积极开展大气污染物源解析，强力实施“利剑斩污”行动，最大限度降低了污染物峰值浓度。2016年PM2.5平均浓度较2013年下降35.7%，空气质量优良率提高35.2个百分点。高度重视水环境安全，加强饮用水源地保护，强化工业排水监管，实施滹沱河、洨河、磁河、汪洋沟综合整治，出境断面水质达到省考核标准。大力开展造林绿化，森林覆盖率达到38.7%，城市绿地率达到40.7%，成功创建国家森林城市和全国绿化模范城市。

人民幸福指数持续提升。坚持更多的财力向民生倾斜，四年累计支出1997.8亿元，占一般公共预算支出的79.9%。社会保障体系不断完善。连续调整提高退休人员和城乡居民养老金待遇，城乡低保实现应保尽保。城镇新增就业41.4万人，城镇登记失业率控制在4.5%以内，农村劳动力转移就业22.2万人。建设公共租赁住房3.94万套。全面打响脱贫攻坚战，23.8万人实现稳定脱贫。社会公益事业全面发展。所有县（市、区）全部通过国家义务教育基本均衡督导评估，义务教育均衡发展取得阶段性成果。新改扩建幼儿园519所。主城区公办学校小学生免费托管服务全覆盖，中职教育资源整合取得突破性进展。山区教育扶贫工程深入实施，累计转移安置学生5.9万名。大力实施“三医联动”，积极开展分级诊疗，扎实推进“医养结合”，被评为“全国幸福家庭创建活动示范市”。公共文化服务体系不断健全，市美术馆、丝弦剧院等文化设施相继投用，《安娥》《太行谣》等一批精品力作荣获全国性奖项。社会治理方式不断创新。加强社会治安防控体系建设，完善社会矛盾纠纷多元化解机制；坚持不懈狠抓安全生产、公共安全和食品药品安全，社会大局保持和谐稳定。举全市之力抗击“7·19”特大洪灾，按照时间节点高标准完成了各项重建任务。国防动员、双拥共建进一步加强，荣获“全国双拥模范城”八连冠。妇女儿童、民族宗教、外事侨务、防震减灾、残疾人、气象、档案、人防等各项事业都取得了新成就。

政府自身建设不断加强。严格落实中央“八项规定”，坚决反对“四风”，扎实开展群众路线教育实践活动、“三严三实”专题教育和“两学一做”学习教育，工作作风明显好转。自觉接受市人大法律监督、市政协民主监督和社会舆论监督，累计办理人大代表建议1831件、政协委员提案2321件，按时办结率达到100%。切实加强政府系统党风廉政建设，不断加大反腐倡廉力度，查处损害发展环境案件1027件，实施效能责任追究653人，为推动省会经济社会健康发展创造了良好环境。

各位代表！四年来，我们紧紧扭住发展第一要务，有效应对经济下行的严峻挑战和不利影响，经济和社会事业发展取得了新成就。这是省委、省政府和市委正确领导的结果，是全市广大干部群众共同奋斗的结果。在此，我代表市政府，向全市人民，向人大代表、政协委员，向各民主党派、工商联、人民团体和社会各界人士，向驻石解放军、武警官兵和政法干警，向中直、省直驻石单位，向关心支持石家庄发展的国内外朋友，致以崇高的敬意和衷心的感谢！

在肯定成绩的同时，我们也清醒地认识到改革发展中存在不少差距和问题，突出表现在“五个不相适应”。一是经济实力与省会地位不相适应，经济总量还不够大，在全省的首位度还不够高，辐射带动能力还不够强，与全国先进省会城市的差距比较明显。二是产业水平与转型升级的要求不相适应，产业结构还不尽合理，传统支柱产业质量效益不高，战略性新兴产业和现代服务业尚未形成有效支撑；改革创新驱动发展的动力不强。三是城镇建设与现代省会定位不相适应，城市承载能力不强，城市景观容貌不美，智慧城市建设滞后，主城区交通拥堵问题比较突出；县城建设规模小、水平低，拉动县域经济发展的能力不强。四是环境治理与人民群众期盼不相适应，大气污染问题突出，2016年PM2.5平均浓度较2015年不降反升，全省污染最严重的30个县市中我市占16个，治污减排任务十分艰巨。五是干部队伍素质与改革发展任务不相适应，一些政府部门和工作人员依法行政、勤政为民的意识还不够强，开拓创新、攻坚克难的能力还不够高。我们一定高度重视这些问题，下大力加以解决。

二、今后五年目标任务

未来五年，是全面建成小康社会的决胜时期，是建设现代化省会城市的关键时期。展望发展大势，全球经济增长低迷态势仍在延续，区域竞争日趋激烈，我们面临着转型升级、加快发展的艰巨任务，面临着经济下行和污染治理的双重压力。同时，我们也面临着难得的发展机遇。京津冀协同发展战略的深入实施，特别是河北雄安新区的设立，将有利于我们加快与京津的准确对接，推动与雄安新区的错位发展；省委、省政府空前重视并大力支持省会建设发展，有利于我们提高综合竞争实力，增强辐射带动功能。刚刚结束的市第十次党代会提出了建设“现代省会、经济强市”的奋斗目标，描绘了石家庄未来五年发展的宏伟蓝图。只要我们紧紧抓住发展机遇，强化使命担当，自觉奋发作为，就一定能够开创石家庄更加美好的未来！

今后五年政府工作的指导思想是：高举中国特色社会主义伟大旗帜，以马克思列宁主义、毛泽东思想、邓小平理论、“三个代表”重要思想、科学发展观为指导，深入学习贯彻习近平总书记系列重要讲话精神和治国理政新理念新思想新战略，紧紧围绕“五位一体”总体布局和“四个全面”战略布局，认真落实省第九次党代会和市第十次党代会提出的目标任务，以新的发展理念为引领，以提高发展质量和效益为中心，以供给侧结构性改革为主线，坚持稳中求进工作总基调，牢牢抓住京津冀协同发展重大历史机遇，大力推进新型工业化、信息化、城镇化和农业现代化，全面深化改革，不断扩大开放，着力推动转型发展、绿色发展、创新发展、率先发展，坚定不移推进全面从严治党，全面建成小康社会，为建设现代省会、经济强市而奋斗。

建设现代省会、经济强市，是第十次党代会确定的今后五年经济社会发展的总目标。我们将围绕建设“产业层次高、城市品质高、文明程度高、幸福指数高”的现代省会，围绕建设“经济实力强、创新动力强、改革活力强、带动能力强”的经济强市，围绕实现“六个显著提升”具体目标，集中力量在以下七个方面攻坚突破：

（一）加快推进动能转换，建设全省经济中心。深化供给侧结构性改革，大力推进产业结构调整，加快构建现代产业体系。发展壮大战略性新兴产业，实现规模翻番，高新技术产业占规上工业的比重达到27%以上；改造升级传统产业，推动传统产业向中高端迈进；加快发展现代服务业，推进生产性服务业向专业化、高端化发展，生活性服务业向精细化、品质化发展；深入推进农业供给侧结构性改革，促进农业向高端特色、高效安全、高产生态转变。集中力量建设一批重大产业支撑项目，做大做强一批品牌园区。到2021年，省会首位度明显增强，生产总值占全省的比重达到20%以上，服务业占GDP的比重达到55%左右，发展质量和效益明显提高，为早日实现万亿GDP打下坚实基础。

（二）强化人才支撑引领，建设全省科技中心。充分发挥高端产业领军人才的支撑引领作用，实施更加优厚的人才政策，广泛吸引海内外高层次人才来我市创新创业，培育和引进一批入选国家“千人计划”“长江学者”和省“百人计划”的高端人才；同时挖掘培养使用好本地人才，鼓励企业自主引进人才，建设人才聚集高地。大力推进协同创新，深度融入京津冀协同发展，在对接和服务京津中借力发展自己。实施高新技术企业、科技型中小企业和创新型企业培育计划，打通产学研融合、军民对接转化、市场化运作“三个通道”，推进产业链、创新链、资金链、政策链“四链”融合。力争五年引进和培养100名创新创业领军人才，高新技术企业突破1000家，科技型中小企业达到2万家，设立研发机构的规上企业达到80%，科技进步贡献率突破60%。

（三）营造良好金融环境，建设全省金融中心。大力发展开放式金融，规划建设金融创新开发区，加速金融机构、金融资产、金融人才等金融要素向我市聚集；积极发展大数据金融、绿色金融、科技金融、互联网金融等新型金融业态；鼓励金融机构创新金融产品，以金融创新推动产业转型，支持地方经济发展。大力推进直接融资，扩大股权、债券、票据和基金融资规模，将石家庄股权交易所打造成全国一流的区域性股权交易市场。到2021年，各类资本市场挂牌上市企业达到260家以上，金融业增加值占GDP的比重达到9%以上。

（四）繁荣发展文化事业，建设全省文化中心。深度挖掘石家庄历史文化资源，打造新乐伏羲、古中山国、正定古城文化品牌，塑造城市独特的历史文化标识。按照公益性、均等性要求，建设一批标志性文化基础设施，完善覆盖城乡的公共文化服务体系。擦亮“彩色周末”等一批文化惠民品牌，创作一批特色文艺精品。广泛开展全民阅读活动，打造书香石家庄。加快发展文化产业，打造滹沱河历史文化与文体休闲产业带、西部红色文化与生态旅游产业带。到2021年，文化产业增加值占GDP的比重达到6%以上。大力发展体育事业，广泛开展全民健身活动。以创建全国文明城市为契机，不断提升市民综合素质和城市文明程度。

（五）突出重点提升水平，建设全省商贸物流中心。完善商贸物流业发展规划，打造全国现代商贸物流重要基地和非首都功能疏解商贸市场重要承载地。科学布局，完善功能，加快主城区批发市场、货运市场、客运枢纽站外移搬迁。加强商贸物流业基础设施建设，推进公铁联运智慧物流枢纽、华北空港货运中心、大型商贸中心建设。主动承接北京商贸企业、批发市场转移，培育国际商贸物流产业集群。加快商贸物流标准化、信息化建设，提升商贸物流智慧化水平。

（六）着力改善环境质量，建设生态化宜居城市。坚持科学治霾、协同治霾、铁腕治霾，加强顶层设计，制定空气质量达标总体规划，大力调整能源结构、工业结构、园区结构和产业布局结构，大幅减少燃煤污染、工业污染、扬尘污染和机动车尾气污染，力促空气质量明显改善，到2021年，PM2.5浓度降至每立方米68微克以下。加强水源涵养，强化水污染治理，全面落实“河长制”，确保主要河流出境断面水质稳定达标。全面开展湿地恢复建设，促进水生态总体改善。严格落实国家“土十条”行动计划，促进土壤资源永续利用。大力开展植树造林，实施矿山复绿工程，构建多级生态绿地体系，到2021年，森林覆盖率达到42%以上，城市绿地率达到41.5%。全面落实生态环保“党政同责、一岗双责”，健全完善环保“网格化”监管体系，坚决打好打赢环境污染防治攻坚战、持久战。

（七）持续增进民生福祉，建设人民满意的幸福城市。贯彻以人民为中心的发展思想，不断增强人民群众获得感，确保城乡居民收入增速高于经济增速。坚决打赢脱贫攻坚战，到2020年确保现行标准下农村贫困人口全部脱贫，在奔向全面小康的道路上绝不让一个贫困群众掉队。推进全民创业增收，创造更多就业岗位，让老百姓的钱袋子鼓起来。提高社会保障标准，增强社会保障能力，让老百姓生活得更加放心。全力办好人民满意的教育，基本建成高水平、优质均衡的现代化教育体系，把我市建设成教育强市和人力资源强市，让更多孩子成就梦想，让更多家庭实现希望。完善公共卫生服务体系，全面提高公共医疗服务水平，让“健康梦”托起“小康梦”。创新完善社会治理，加强源头治理、基层治理、专项治理，打造“最平安省会城市”，让人民群众生活得更有安全感。

各位代表！规划未来是为了创造未来。今后五年，我们将发扬“钉钉子”精神，一锤接着一锤敲，努力把蓝图变为美景，把目标变为现实，绝不辜负全市人民的重托和希望！

三、2017年重点工作

今年是贯彻省第九次党代会、市第十次党代会精神的开局之年，是落实“增强辐射带动功能，加快建设现代化省会城市”新定位的起步之年，我们将迎来党的十九大胜利召开。做好今年的工作，具有特殊而重大的意义。我们必须坚持稳中求进工作总基调，把“聚焦新定位、聚力新路径、展示新作为、开创新局面”作为基本遵循，把今年作为省会建设发展的“攻坚突破年、提质提升年、改革创新年”，力促省会经济社会各项事业又好又快发展。

主要预期目标是：生产总值增长7.5%左右，其中规模以上工业增加值增长6%，服务业增加值增长10.5%；一般公共预算收入增长8%；固定资产投资增长10%，社会消费品零售总额增长10.2%；城乡居民人均可支配收入分别增长8%，居民消费价格指数涨幅控制在3%左右。重点做好以下八个方面的工作：

（一）大力振兴实体经济，打造产业转型升级示范区。着力改善供给侧结构，坚持高端化、智能化、规模化方向，不断提高经济发展的质量、效益和综合竞争力。

深入推进“三去一降一补”。严格执行环保、能耗、质量、安全等规定和标准，确保压减炼铁产能52万吨、水泥产能350万吨、焦炭产能80万吨，有序淘汰30万千瓦以下纯凝发电机组，限期关停违法违规建设的燃煤自备电厂。加强房地产市场分类调控，确保商品办公类用房库存消化周期控制在合理区间。认真落实去杠杆政策措施，有力有序推进资产证券化和债转股，加大股权融资力度，稳妥推动“僵尸企业”重组或退出市场。大力推进减税降费，让企业轻装上阵。聚焦县域经济这个最大短板，大力实施“一二三四”县域经济发展战略，培育壮大主导产业，加快形成产业集群，争取全国百强县“零”的突破，坚决打赢县域发展翻身仗。

培育壮大新兴产业。围绕高端装备制造、生物医药、新一代信息技术、节能环保等战略性新兴产业，加快推进通用航空、光电导航等产业基地建设，谋划建设空气净化产业园，重点抓好常山云数据中心等89个重大项目。大力发展智能制造，力争在人工智能、工业机器人、第五代移动通信等高端领域，实现“从无到有”的突破、“从有到强”的跨越。鼓励发展分享经济、数字经济等新兴业态，提高社会资源利用效率，促进“互联网+”经济加快成长。

改造提升传统产业。落实“中国制造2025”战略，应用大数据、云计算、物联网技术，对食品、纺织等传统优势行业进行绿色和智能化改造，滚动实施重点技改项目100项以上。大力推进工业化和信息化深度融合，培育一批省级两化融合示范企业。加快发展各类中小微企业，推动“个转企、小升规、规改股、股上市”，年内新增规模以上工业企业120家以上。继续开展入企帮扶活动，破解企业发展难题，支持优势企业做大做强。弘扬企业家精神和工匠精神，开展消费品工业“三品”专项行动，加强质量管理，打造品牌企业、名牌产品。

加快发展现代服务业。落实好我市支持服务业发展的20条政策，推动现代服务业发展提速、比重提高、层次提升，力争服务业占GDP的比重达到49%。一是围绕打造全国现代商贸物流重要基地，规划建设主城区和正定新区中央商务区，推进藁城内陆港物流园等10大物流园和华润万象城等5大商贸综合体建设。二是围绕建设国家电子商务示范城市，推进金融电商示范基地等园区和平台建设，促进电商、快递进社区进农村。三是围绕打造全省金融中心，依托自强路金融街区建设金融创新开发区，引导鼓励更多的银行、保险、证券等金融机构入驻省会，全市金融业增加值达到460亿元，增长10%以上。四是围绕建设旅游强市，编制石家庄全域旅游和智慧旅游规划，支持平山、正定、鹿泉争创国家级全域旅游示范区，支持赞皇、井陉、灵寿争创省级全域旅游示范区。加快环省会旅游带建设，重点打造君乐宝乳业小镇等10个特色旅游小镇。办好首届旅发大会暨第四届旅交会，打响石家庄旅游品牌。旅游业总收入增长25%以上。五是围绕建设康养休闲基地，推进富力健康城等5大健康城建设，加强与京津健康养老服务机构对接合作，促进医养融合发展。六是围绕发展会展经济和体育产业，办好石洽会、动博会、正博会、国际通用航空博览会等30个重点展会，组织好马拉松赛、国际自行车环城赛、中甲足球赛等14项重要体育赛事。

强力推进重点项目建设。实施“重大项目建设转型升级年”活动，今年安排市重点项目300项，确保高端大功率芯片等100个项目竣工，四药生物制药园等100个项目开工，力争博鹰通航等100个项目签约落地。对近三年来重点项目建设情况，进行盘点验收，逐一摸清底数，制定帮扶措施，力争早日投产见效。扎实做好项目谋划储备工作，确保全市亿元以上项目保持在1500项以上。完善项目建设推进机制，实行领导分包、约谈通报等制度，定期开展项目观摩拉练和集中开工活动，强化市直部门与县（市、区）捆绑考核，确保项目建设顺利推进。着力解决项目建设土地制约瓶颈，创新征地工作思路，提高新增建设用地执行率；加快平山、行唐、灵寿、赞皇、井陉等山区县岗坡次地开发，加大土地整理力度，增加造地面积，解决土地占补平衡问题，加快项目落地速度。

狠抓开发区增比进位。理顺开发区管理体制和运行机制，深化机构编制改革、人事薪酬改革、行政审批改革、投融资体制改革，推行“市场化运作、企业化经营”机制，引进国内外战略投资者、专业化园区运营商，力争在整体外包、特许经营上实现“破冰”。高标准建设基础设施、配套设施和公共服务平台，年内所有省级以上开发区全部达到“九通一平”，两个国家级开发区率先实现新“九通一平”。加大开发区招商引资力度，从政府主导向政府招商与市场化招商相结合转变，集中力量引进2-3个产业链核心项目，加快培育优势特色产业集群，创建一批品牌化园区。确保开发区主要经济指标增幅明显高于全市平均水平，力争2个以上开发区进入省级新型工业化产业示范基地。高新技术开发区和经济技术开发区主营业务收入分别突破3000亿元、1500亿元。

产业兴则经济强，我们必须向产业要实力，把转型发展作为省会发展的第一要求，努力跑出省会经济发展的“加速度”！

（二）强力整治环境污染，推进生态环境持续好转。强化“生态优先、绿色发展”理念，以重任在肩、寝食难安的责任意识，以壮士断腕、决战决胜的坚定信念，全力实施“五大行动”。

实施“蓝天”行动。全面贯彻落实省委、省政府《关于强力推进大气污染综合治理的意见》以及18个专项实施方案，集中力量抓好四大攻坚，强化两大支撑，坚决打赢蓝天保卫战，确保PM2.5年均浓度下降20.2%，降至每立方米79微克。四大攻坚：一是燃煤压减替代攻坚。10月底前完成主城区和鹿泉、正定、藁城、栾城39万户气代煤、电代煤任务。全面淘汰10蒸吨以下燃煤锅炉和市区建成区35蒸吨以下燃煤锅炉。完成石热九期燃气机组和西柏坡电厂余热入市工程建设任务，加速实现主城区无煤化。二是扬尘综合治理攻坚。年底前关闭20家露天生产矿山，今冬明春完成92家责任主体灭失矿山复绿任务，砂场控制在26家以内。建筑工地全部安装在线视频监控。全面落实“以克论净”管理制度，提高道路扬尘治理精细化管理水平。严格渣土车管理，确保全部车辆安装密闭装置并正常使用。三是机动车尾气治理攻坚。9月底前力争全面供应国六车用汽柴油。在全市主要路口加装遥感监测设备，建设联合执法工作站，加强对营运车辆的监控。规划实施307国道等公路改扩建工程，完善过境交通网络，解决主城区和井陉、鹿泉、藁城货运车辆穿城过境、扬尘和尾气污染问题；年内采取“远端绕行、区内严控”交通管制措施，严格分流管控穿城过境货运车辆。四是工业污染治理攻坚。9月底前重点行业全部达到特别排放限值要求。实现重点企业在线监控全覆盖。10月底前，基本完成石油化工、医药农药、汽车修理、家具制造等重点行业挥发性有机物（VOCs）综合治理。按照“有保有压、一企一策”的原则，科学组织企业错峰生产。实施钢铁、水泥、建筑陶瓷等8大行业调整提升工程，制定焦化行业有序退出计划，关停污染企业400家以上，整治提升150家以上。同时，全面取缔“散乱污”企业。两大支撑：一是技术支撑。建设智慧环保大数据平台，通过大数据和云计算，实现对重点行业和重点企业的实时监控，为大气污染防治提供技术和智力支撑。二是资金支撑。积极争取上级专项资金，最大限度用好国家政策。建立大气污染防治投融资平台，用好国开行中长期低息贷款。发挥股权投资基金的拉动作用，大力推广PPP、环境污染第三方治理等模式，通过市场化运作撬动社会资本参与大气污染治理。

实施“碧水”行动。围绕治污水、保供水、抓节水，实施岗南水库、黄壁庄水库上游导污治污滹北渠更新改造工程，防范上游水污染环境风险。高标准实施滹沱河市区段至深泽段整治工程，把省会的母亲河建设成为最靓丽的生态景观带。完成各县（市、区）污水处理厂改造提升工作，污水排放全部达到一级A标准。严格落实河流跨界断面水质目标考核责任制，确保跨界断面水质达到省考核标准。全面治理城市黑臭水体。建立健全水价调整补偿机制，推进地下水超采综合治理，完成省确定的压采任务，促进水生态修复。

实施“净土”行动。出台土壤污染防治计划，初步建立土壤污染防治体系。严格实施场地环境调查、风险评估、治理修复，保障工业企业场地再开发利用的环境安全。有序推进土壤污染治理与修复试点示范，抓好县域土壤污染治理与修复示范项目。做好全市农用地土壤污染状况调查工作，完善土壤污染治理与修复项目数据库。

实施“绿地”行动。落实京津冀生态环境支撑区规划，全面推进生态绿化工程建设，深入实施环城绿化、西部太行山区绿化、北部“三河”绿化、绿色通道提升等四大工程，增加全市森林资源总量，完成人工造林40万亩、封山育林30万亩，新增城市绿地900万平方米，森林覆盖率达到39.9%。

实施“雷霆”行动。加强联防联控，提高县域、城乡接合部管理水平，确保市县同步采取最严格治理措施。开展联合执法、区域执法、交叉执法、错时执法，严格执行按日计罚、查封扣押、限产停产、行政拘留等法律规定，保持对环境违法犯罪的严打高压态势。全面开展环保督察，既督企，又督政，切实加大监管力度。设立环境污染举报专线，实行有奖举报，形成全民参与、全民监督的浓厚氛围。

绿色发展是省会发展的第一选择，改善生态环境是最迫切、最重大的民生工程。我们一定科学施策、标本兼治，打好环境污染综合治理的人民战争，让石家庄的天更蓝、地更绿、水更清！

（三）深度融入京津冀协同发展，开创省会发展新局面。京津冀协同发展是我们加快发展的重大机遇。设立雄安新区，是千年大计、国家大事，必将开启新一轮改革开放发展的新征程。

全方位开展对接合作。以建设石保廊全面创新改革试验区为契机，重点抓好与中关村、亦庄开发区的合作，全力推进中关村（正定）集成电路产业基地、正定科技新城、北京协同创新园石家庄分园等项目建设。主动吸引京津“双一流”高校、知名三甲医院等机构，与我市开展深度合作。充分发挥京津冀产学研联盟和科技大市场作用，构建“京津研发+石家庄转化”的创新格局。瞄准京津市场需求，把石家庄建成京津冀重要的农产品供应基地、养老休闲基地。

大力度建设承接平台。把正定县（正定新区）、国家级和省级开发区作为承接京津产业转移的重要平台，积极引进京津现代产业和优势企业，加快培育一批优势特色产业集群。重点加强正定县（正定新区）基础设施建设，提升承载能力。推动正定新区及早纳入天津自贸区政策范围，打造融入京津冀协同发展的战略支点。

构建一体化交通网络。统筹铁路、公路、航空等交通方式，抓好京石城际、石邯城际、石衡沧城际、津石客专、石太客专等铁路的谋划建设，加快推进火车东站及广场建设，确保与石济客专同步投用。加快南绕城高速建设，力争明年竣工通车；推进平赞、西阜高速建设，今年完成工程总量的45%以上；做好津石、石衡高速前期工作，力争年内开工。加快正定机场、栾城通用航空机场建设，构建综合立体交通枢纽，实现与京津和周边城市的互联互通。

（四）加快新型城镇化进程，打造高品质省会城市。以建设国家新型城镇化综合试点城市为契机，瞄准“增强辐射带动功能，建设现代化省会城市”新定位，全方位提升城镇规划建设管理水平。

加快修编城镇规划。坚持以高水平规划引领城市发展，以先进理念构建城镇体系，优化空间布局，提高城市品位。着眼建设新型城镇化与城乡统筹示范区，科学确定城镇的规模结构和功能定位。按照“全域统筹、多规合一”的理念，以总体规划修编为契机，编制海绵城市、综合管廊、通风廊道、综合交通、地下空间利用等专项规划，制定中央商务区、西部山前区、正定古城等城市重点功能分区规划。推进新区和古城融合发展。强化鹿泉、正定、藁城、栾城与主城区的产业承接、交通连接、功能衔接、生态对接，尽快实现同城化发展。支持晋州、新乐、平山建设市域次中心。

打造立体交通路网。规划建设主城区通往鹿泉、正定、藁城、栾城、重大产业园区和西部景区的轻轨，今年做好前期准备工作。确保轨道交通1、3号线一期首开工程安全有序运营，加快1号线二期、2号线一期、3号线一期两边段及二期建设。完成南二环东西延、和平路高架西延、槐安路友谊大街匝道、槐安路东二环立交完善等工程。实施中华大街南延等断头路打通工程，年内打通5 条以上。启动新城大道北延、中华大街北延等项目。开工建设华南路等40条支路。加快公交都市建设，谋划建设快速公交系统，新建30座港湾式停靠站，增加公交专用道90公里。启动环城快速路前期工作。

提升城市载体功能。加大滹沱河生态修复力度，打造“城市之眼”。推进龙泉湖湿地建设，同步实施西部山前区基础设施提升工程。新建西环公园等5座综合性公园、15万平方米街旁游园。实施小清河综合治理工程，大幅改善城区西北部居住环境。建设宁安路、康乐街等雨污分流工程。5月底前完成南水北调配套水厂水源切换，全年江水用量达到规划分配水量的40%以上。实施塔北路、仓丰路等地下综合管廊工程，完成45.7公里建设任务。抓好海绵城市建设。加强防灾减灾救灾体系建设，提升综合防范能力。

强力推进新区建设。大力实施城市北跨发展战略，高起点、高站位完善正定县（正定新区）总体规划，加快建设地上轻轨、综合管廊等基础设施。重点抓好30平方公里核心区建设，全力推进奥体中心、国际展览中心、规划馆、图书馆、青少年活动中心等功能性场馆建设。规划建设高校聚集区，加快职教园区建设，确保市交通运输学校、信息技术学校（二期）等4所学校顺利开工。继续抓好正定古城保护，高标准推进24项重点工程，尽快实现古城环境风貌的大幅跃升。

提高城市管理水平。加快智慧城市建设，编制智慧城市建设规划，以智慧环保建设为突破口，整合相关部门信息技术数据，加快智慧交通、智慧城管、智慧社区、智慧政务建设，年内在正定新区综合商务中心建成高标准“智慧大厅”，让居民享受智慧城市建设带来的便利生活。实施城市综合执法体制改革，提升城市管理水平。主街主路机械化清扫率达到90%以上。坚持疏堵结合，年内主城四区各新建3-5个便民市场，规范清理占道市场。加强对重点片区的规划管控，以土地大片区收储为抓手，做活城市经营文章，为城镇建设提供支撑。

实施县城攻坚行动。着眼县城环境做美、功能做优，大力推进“三改一拆”行动，强力整治违法、危陋建筑，每个县至少建设1个特色风貌街区、2座精品建筑，综合整治3条景观道路，新乐、高邑等6个县城争创“洁净城市”。晋州、正定积极创建国家园林县城，高邑、矿区争创省级生态园林县城，赵县、无极、灵寿、赞皇、深泽建成省级园林县城。加快县城基础设施和公共服务设施建设，年内所有县城全部实现集中供暖、集中供水和集中治污，增强吸引人口、聚集产业的能力。今年，力争高邑进入全省县城建设前10名，晋州进入全省前20名，正定、元氏进入全省前30名。

加快城乡融合发展。坚持城乡统筹、产城互动，扎实推进以人为核心的新型城镇化。实施特色小镇培育行动，规划建设一批工业强镇、商贸重镇、旅游名镇，努力打造城乡统筹、转移就业、促进增收的新平台。推动城乡基本公共服务均等化，通过户籍制度、居住证制度、就地城镇化等方式，促进农业转移人口市民化，全市常住人口城镇化率达到61%。

推进省会率先发展是我们的第一目标。我们一定奋发图强，全力打造一个布局合理、功能完善、魅力彰显、舒适宜居的现代化省会城市！

（五）拓展改革开放深度广度，释放发展动力和活力。坚持靶向原则，突出问题导向，加大关键环节改革力度，提升对外开放水平，进一步释放改革开放红利。

持续推进“放管服”改革。深化行政审批制度改革，落实改善营商环境的23条意见，编制市县两级行政许可通用名录和公共服务专项清单，在县级全部实行项目环评备案制。开展市直机关内设机构改革和精简人员编制工作，提高行政管理效能。全面落实权力清单、责任清单、监管清单和负面清单管理制度，减少政府自由裁量权，增加市场自主选择权。

继续深化商事制度改革。全面实施“多证合一”“一照一码”，实行全程电子化登记管理。完善事中事后监管制度，“双随机、一公开”抽查机制实现全覆盖。建立全市“一张网”的涉企信息互通共联机制。推进企业登记注册、注销便利化，规范中介服务和收费，激发市场主体活力。

深入推进财税金融改革。推进市、县两级财政事权和支出责任划分，做好“营改增”后续工作。抓好绩效预算管理改革，建立专项支出动态绩效指标。市财政注入1亿元，建立“工业企业稳定发展资金池”。加大PPP项目谋划储备力度，推进项目更多更早落地实施。实行政府投资项目代建制，统筹建设项目，保障工程质量，提高投资效益。支持具备条件的民间资本发起设立民营银行、民间资本管理公司。

加快推进国企国资改革。改善和加强国有资产监管，将市级机关事业单位所属企业的国有资本，逐步纳入经营性国有资产，实现集中统一监管。全力推动国有企业“三供一业”分离移交，解决历史遗留问题，推进国有企业瘦身健体。坚持权利平等、机会平等、规则平等，进一步放宽非公有制经济市场准入，鼓励非公有制企业参与国有企业改革。

深化农业农村改革。完善农村土地“三权分置”办法，落实集体所有权，稳定农户承包权，放活土地经营权，年内农村宅基地和集体建设用地确权登记发证工作完成30%。稳步推进农村集体产权制度改革，抓好27个农村股份制改造试点。继续深化供销社综合改革，打造政府用得上、农民信得过的综合服务平台。启动农业水资源税改革工作，年底前全面完成农业生产超限额水量水资源税征收工作。

大力推进双向开放。推动更高水平“引进来”、更大步伐“走出去”，积极融入“一带一路”战略，扶持水泥、钢铁、机械装备等传统优势企业，与中东欧、拉美、东盟、非洲开展国际产能合作。着力构建“北接”“西拓”“东出”“南联”区域开放格局，拓展对内开放空间。抓住河北电子口岸正式运营的机遇，加快综合保税区建设，实现跨境电商在省会的率先发展。全年重点培育10家跨境电商示范企业、3个跨境电商平台，支持企业建设跨境电商公共海外仓。实施专业化、精准化招商，建立工作台账，加大考核奖惩力度，确保招商引资取得实效。全年实际利用外资增长5%。

（六）深入实施创新驱动战略，提升自主创新能力。围绕创建国家创新型城市，加大创新力度，营造良好创新生态环境，推动大众创业、万众创新。

壮大创新创业主体。大力培育创新型企业，在人才引进、政策扶持、配套建设上提供量体裁衣式服务，促进企业从“模仿创新”向“集成创新”“原始创新”转变，全年新认定高新技术企业180家。继续实施科技型中小企业成长计划，推进苗圃工程、雏鹰工程、科技小巨人工程，确保科技型中小企业发展到8500家，力争突破1万家。发挥企业科技创新的主体作用，鼓励所有规上企业开展研发活动，年内设立研发机构的规上企业达到23%。

强化创新平台建设。鼓励企业与科研院所开展合作，加快企业技术中心、重点实验室等创新平台建设，新培育认定市级以上创新平台30家。推进军民深度融合发展，加快军民融合产业园建设。加强科技资源共享服务平台、大型仪器设备共享平台、网络服务平台以及云平台建设，提高科技服务水平。建设一批孵化器、加速器、科技型中小企业“园中园”，促进科技成果落地转化。

充分激发“双创”活力。全面落实《关于引进高端产业人才的意见》，实施高精尖人才培养引进工程，千方百计吸引高端产业领军人才来我市创新创业。年内引进50名以上科技型创新英才，引进和培养20名以上创新创业领军人才。加快建设一批“双创”基地，鼓励大企业、科研院所、高校设立专业化众创空间，年内新认定20家以上。

创新发展是省会发展的第一动力。我们必须把创新摆在发展全局的核心位置，让创新成为省会发展的最强引擎！

（七）切实抓好“三农”工作，大力发展现代农业。一般农产品过剩、优质农产品短缺，是制约农业增效、农民增收的关键。必须以农业供给侧结构性改革为主线，建设现代农业园区和智慧农业，推进农业绿色生产、农村绿色生态、农民绿色生活，不断增加农民收入。

优化农业生产布局。以建设国家现代农业示范区为引领，积极打造“一圈两区”现代农业发展新格局。鹿泉、正定、藁城、栾城加快发展休闲观光农业，打造环省会都市农业圈；平原县大力发展“绿色优质农产品生产基地+中央厨房”，打造高水平的现代农业示范区；山区县大力发展“经济沟+生态农业”，建设特色农业优势区。年内建设3-5条特色农业观光线路，重点培育10个以上要素聚集、装备先进、链条完整、带动力强的现代农业园区。

推进农业结构调整。在确保粮食安全的基础上，调优、调特、调强种养结构，扩大强筋麦等优质专用小麦生产，适度调减籽粒玉米种植面积，加快发展药用作物、食用菌、小杂粮等特色产业，增加优质绿色农产品供给。实施“一县一品一牌”培育计划，建立健全农业标准化生产体系、农产品质量追溯体系和质量安全检验检测体系，年内无公害标准化蔬菜、果品种植面积分别达到230万亩、240万亩，标准化规模养殖示范场达到978家，培育一批具有石家庄特色的地理标志品牌。

壮大农业产业化规模。依托农业龙头企业，大力发展农产品精深加工，促进农村一二三产业融合发展，农业产业化经营率达到67.5%以上。加快培育新型农业经营主体和农业产业工人，发展适度规模经营，家庭农场发展到1600家，适度规模经营率达到30%。加快智慧农业建设，抓好藁城智慧农业试点，实施农业物联网示范工程，推进支农惠农信息进村入户。

加快美丽乡村建设。按照“四美”要求，实施民居改造、安全饮水、街道硬化等十二个专项行动，抓好平山西柏坡、井陉古村落、藁城休闲旅游体验等6个省级重点片区，完成353个省级重点村建设，打造50个以上精品村。同时，继续下大力抓好“7·19”水毁工程修复，确保按时完成7个县、639个村的农村饮水工程灾后重建任务，解决35.26万灾区群众饮水安全问题。

加大脱贫攻坚力度。坚持精准扶贫、精准脱贫，紧紧围绕“两不愁、三保障”，扎实推进“七个一批”专项行动和基础设施建设“七大工程”，结合现代农业发展、美丽乡村建设、山区综合开发和太行山生态绿化、乡村旅游，大力发展脱贫产业，引进股份合作机制和龙头企业，持续增加农民收益。全面启动易地搬迁扶贫工程，解决好就业、医疗、社保、子女就学等问题，确保“搬得出、稳得住、能致富”。突出抓好革命老区贫困村的脱贫工作，改善老区群众生产生活条件。制定鼓励返乡创业的优惠政策，支持各类人员返乡创业，促进扶贫、创业相结合，带动农民持续增收。确保全年165个贫困村、4.6万人稳定脱贫。

（八）顺应人民群众期盼，尽心竭力保障和改善民生。坚持民生优先、民生先动，加大民生投入，全力办好民生实事，不断增强人民群众获得感。

扎实做好就业再就业工作。认真贯彻落实化解过剩产能和环境治理援企稳岗政策，对失业人员进行特别职业培训。实施就业促进、创业引领、基层成长等行动计划，组织开展就业援助、春风行动、民企招聘等专项活动，促进大学毕业生多渠道就业创业。全年城镇新增就业10.3万人，城镇登记失业率控制在4.5%以内，农村劳动力转移就业5.15万人。

织密扎牢社会保障网。实施全民参保计划，扩大社保覆盖面，确保城镇职工基本养老保险参保人数达到228.6万人。积极推进机关事业单位养老保险制度改革。提高农村低保标准，实现低保线与扶贫线“两线合一”。提升养老服务、社会救助能力，搭建社区养老服务平台。做好住房保障工作，建成保障房8000套，棚户区改造开工建设2.18万套。

努力办好公平优质教育。抓好普惠性学前教育，推进义务教育优质均衡发展，提升高中教育质量。深化学区管理制和名校集团化办学体制改革，启动实施消除大班额专项规划。继续推进山区教育扶贫工程，落实建档立卡贫困学生资助政策。加快构建现代职业教育体系，完善校企合作、工学结合的人才培养机制。优化市属高校专业结构，提高服务地方经济发展的水平。

推进健康石家庄建设。健全完善公共卫生服务体系，深化医药卫生体制改革，启动城市公立医院综合改革，全面取消药品加成，公立医院综合改革示范县创建实现全覆盖。推进国家中医药综合改革试验市建设，创建8家全国基层中医药工作先进单位。加快市一院赵卜口新院区建设，确保市四院谈固新院区等竣工使用，抓好与北京、天津合作医院签约项目落地。深化计划生育服务管理改革，加强妇幼健康服务保障。

推动文化体育事业繁荣发展。建立健全城乡公共文化服务体系，深入实施文化精品、文化惠民、产业提升“三大工程”，提高基本公共文化服务均等化水平。加大文化基础设施投入，谋划建设博物馆新馆等文化设施。大力推进国家文化消费试点城市建设，推进河北东方文化创意产业基地、河北新东印刷等聚集区建设，扩大文化消费。大力发展体育事业，加快全民健身公共服务体系示范区建设，高标准完成省运会筹备工作。

提高社会治理水平。完善社会矛盾纠纷排查化解机制，加强重点行业安全监管和重点人群服务管理，及时妥善处置各类突发事件。依法规范信访秩序，及时就地解决群众合理诉求。积极创建全国公共安全视频建设联网应用示范市，加强县乡村三级综治中心规范化建设，完善立体化、信息化社会治安防控体系，严厉打击各类刑事犯罪活动。严格落实安全生产责任制，坚决遏制重特大安全事故发生。强化食品药品安全管理，创建国家食品安全示范城市。健全社会诚信体系，建设综合性征信平台。大力支持军队和国防后备力量建设，加强国防教育，深入开展双拥共建活动。积极做好民族宗教、妇女儿童、残疾人、外事侨务、人防、档案、气象、防震等各项工作，促进各项社会事业全面进步。

办好利民惠民十件实事：一是治理城市交通拥堵。大力开展管理治堵和工程治堵，有效缓解交通拥堵问题。二是发展居家养老服务。新改扩建60个综合居家养老服务中心、240个社区居家养老服务中心。三是提高贫困人口医疗保障救助水平。对符合条件的患病贫困人口，给予医疗救助。四是继续改造老旧小区。改造升级市区80个老旧小区的低压电路设备，改造1450万平方米供热面积涉及的老旧管网，分步解决三层以上老旧小区吃水难问题。五是发展普惠性学前教育。新增30所普惠性幼儿园，提供优质普惠的学前教育服务。六是发行文化惠民（演艺）卡。发行2万张文化惠民（演艺）卡，其中向城镇低保家庭免费发放1000张。七是建设完善10分钟健身圈。在城市社区更新安装全民健身路径100条，在农村安装体育健身设施200套。八是实施免费无线网络（WIFI）覆盖工程。省会三环以内公共场所，所有县（市、区）城区公共场所实现免费WIFI覆盖。九是实施医养结合示范工程。每个县（市、区）建立1个标准化医养结合示范机构，50%以上的养老机构能够提供医疗卫生服务，80%以上的医疗机构为老年人开通医疗服务绿色通道，重点人群居家养老家庭医生签约服务率达到100%。十是开通免费律师、公证顾问平台。依托司法行政公共法律服务系统，向广大群众、小微企业推送律师、公证服务。

人民对美好生活的向往，就是我们的奋斗目标。关乎民生的事，事事在心；顺乎民意的事，件件落实。我们一定加倍努力，让全市人民生活的更安康、更殷实、更幸福！

四、加强政府自身建设

面对新形势、新任务，我们必须强化市场思维、法治意识，用创新的办法突破发展瓶颈，用担当的精神破解发展难题，以效能大提升促进省会大发展。

（一）从严治政，创造良好环境。严守政治纪律和政治规矩，牢固树立“四个意识”，自觉向以习近平同志为核心的党中央看齐，向党中央、国务院，省委、省政府和市委决策部署看齐，敬畏职责，勇于担当，站排头、争一流、做表率，确保各项工作走在全省前列。深入推进政府系统党风廉政建设和反腐败工作，严格落实“两个责任” “一岗双责”和中央“八项规定”精神，坚决反对“四风”。强化对人权、事权、财权的制度约束，紧盯重要领域、重要岗位和关键环节，加大行政监察和审计监督力度，严肃查处各种不正之风和腐败问题，以反腐倡廉的实效取信于民。

（二）转变职能，提升政府效率。创新政府管理模式，破除制约发展的思想观念和制度藩篱，坚决砍掉繁琐手续，打通信息孤岛，实现各部门信息互联互通，大力提升公共服务质量和效率。进一步增强简政放权的针对性、系统性、协调性，主动调整下放市级行政权力，推进精准放权、精确承接；全面推进市、县行政审批局规范化、标准化建设，提高审批效率。推进市、县、乡三级实体政务大厅向网上办事大厅转变，最大程度方便群众和企业。加大政府工作人员教育培训力度，提高政治素养、业务水平和履职能力。

（三）依法行政，主动接受监督。严格按照法定权限和程序履行政府职能，做到法定职责必须为，法无授权不可为，确保政府一切工作在法律的框架下进行。进一步健全依法科学民主决策机制，自觉接受市人大法律监督、市政协民主监督和社会舆论监督，认真办理人大代表建议和政协委员提案，主动接受人大执法检查和专题质询，落实重大事项向市人大报告和向政协通报制度。全面实施“七五”普法，提高依法办事能力。深化市县综合执法改革，完善行政执法程序，规范行政执法行为。拓宽政务公开渠道，确保权力规范阳光运行。

（四）实干实政，改进工作作风。坚持把抓落实作为转变作风的重要着力点，定任务、定责任、定时限、定奖惩，大力整顿工作作风，严查不作为的人，紧盯不落实的事，对为官不为、履职不力的严肃问责，对懒政怠政、失职渎职的严厉惩处。贯彻落实正向激励机制和容错纠错机制，调动政府工作人员想干事、敢干事的积极性和主动性。大力开展“双问计”活动。深入一线调查研究，听实话、察实情、出实招、求实效。大兴“马上就办、办就办好”之风，以优良的作风保障各项事业顺利推进。

各位代表！使命重在担当，实干铸就辉煌。让我们更加紧密地团结在以习近平同志为核心的党中央周围，在省委、省政府和市委的正确领导下，紧紧依靠全市人民，坚定信心，攻坚克难，奋发进取，为全面建成小康社会，加快建设现代省会、经济强市而不懈奋斗，努力谱写石家庄更加灿烂辉煌的美好篇章！
